# Impact of *miR-1/miR-133* Cluster in Cardiogenesis and Heart Failure

**DOI:** 10.3390/jcdd13080376

**Published:** 2026-08-07

**Authors:** Carlos García-Padilla, Estefanía Lozano-Velasco, Virginio García-López, Amelia Aránega, Diego Franco, Virginio García-Martínez, Carmen López-Sánchez

**Affiliations:** 1 Department of Human Anatomy and Embryology, Faculty of Medicine and Health Sciences, Institute of Molecular Pathology Biomarkers, University of Extremadura, 06006 Badajoz, Spain; evelasco@ujaen.es (E.L.-V.); garcialopez@unex.es (V.G.-L.); virginio@unex.es (V.G.-M.); 2 Department of Experimental Biology, University of Jaen, 23071 Jaen, Spain; aaranega@ujaen.es (A.A.); dfranco@ujaen.es (D.F.); 3 Medina Foundation, 18016 Granada, Spain; 4 Department of Medical and Surgical Therapeutics, Division of Clinical Pharmacology, Faculty of Medicine and Health Sciences, University of Extremadura, 06006 Badajoz, Spain

**Keywords:** cardiogenesis, *miR-1*/miR-133 cluster, cardiac development, heart diseases, heart failure

## Abstract

Cardiogenesis begins during gastrulation with the bilateral specification of precardiac mesoderm on either side of the embryo. These fields fuse at the embryonic midline to form the linear heart tube, which subsequently undergoes rightward looping and chamber formation. Expansion and septation of the primitive atrial and ventricular chambers establish separate systemic and pulmonary circuits with correctly aligned inflow and outflow tracts. These morphogenetic events depend on the coordinated activation and repression of cell type-specific cardiac gene programmes controlled at transcriptional and post-transcriptional levels by cardiac transcription factors and paracrine/autocrine signals. Non-coding RNAs, particularly microRNAs and long non-coding RNAs, add an essential regulatory layer. MicroRNAs are endogenous ≈22-nt RNAs that modulate gene expression predominantly at the post-transcriptional level. The muscle-enriched *miR-1/miR-133* cluster is essential for normal cardiogenesis, whereas dysregulation of *miR-1* and *miR-133* contributes to heart failure and its associated pathological processes. Here, we provide a state-of-the-art review of the genomic regulation, developmental functions, context-dependent interactions, and translational potential of the *miR-1/miR-133* cluster in cardiogenesis and heart failure.

## 1. Introduction

For decades, scientists have considered non-coding RNAs (ncRNAs) as a non-functional part of the genome, focusing their attention on coding RNA biology. The sequencing of the human genome, and later the ENCODE project, have shown that more than 80% of the genome is transcribed in some type of RNA [1,2] Interestingly, less than 2% of this transcribed genome corresponds to coding RNAs, suggesting that ncRNAs may be as or more relevant than coding RNAs. Currently, it has been demonstrated that ncRNAs are essential for regulation of cellular pathways and biological processes, such as cell development, differentiation, growth, homeostasis and disease [3,4,5,6,7,8,9]. According to their length and characteristics, ncRNAs are classified into (i) short or small non-coding RNAs (≤200 nucleotides), including microRNAs, snoRNAs, piRNAs and tRNAs, (ii) long non-coding RNAs (>200 nucleotides), including subtypes such as intronic, enhancer, circular and intergenic long non-coding RNAs (lncRNAs), and (iii) circular RNA (circRNA), which lacks free ends and comprises a wide range of ncRNAs [10,11,12,13,14].

MicroRNAs are approximately 20–22 nucleotides long and predominantly repress gene expression by complementary base pairing with target RNAs, most commonly within their 3′ untranslated regions, thereby promoting transcript degradation and/or translational inhibition [15,16]. Primary microRNAs are processed by the Drosha–DGCR8 microprocessor, exported through the Exportin-5/Ran-GTP pathway, and cleaved by Dicer; one strand of the resulting duplex is then loaded into an Argonaute-containing RNA-induced silencing complex (RISC) [17,18,19,20,21,22,23]. Beyond this canonical cytoplasmic pathway, nuclear microRNAs can modulate transcription, chromatin state, and transcription-factor activity, although the underlying mechanisms remain incompletely defined [24,25,26,27].

The roles of microRNAs as pivotal modulators of several cellular processes have been widely described, including cellular proliferation and differentiation, maintenance of cellular homeostasis and specification of different cellular lineages [28,29,30]. Likewise, deregulations of microRNAs have been identified associated with various pathologies [31,32,33,34]. In this context, the use of microRNAs as disease regulators has been addressed, highlighting their impact as potential therapeutic molecules [35,36].

Noticeably, many reports have pinpointed the crucial role of microRNAs governing distinct embryonic pathways, including cardiac development, a complex process tightly regulated by several transcriptional and post-transcriptional regulators, so that disturbing embryonic cardiac gene programme results in a plethora of different cardiovascular diseases, many of them non-compatible with the sustaining of adult life [37,38]. In this sense, transcriptional modulation of cardiogenesis is exerted by several transcription factors, such as *Mesp1/2*, *Gata4*, *Nkx-2.5*, *Srf*, *Pitx2c* and *Mef2c* [39,40,41,42,43]; chromatic remodelling factors, such as Hdac2 or Hdac4 [44], and paracrine and exocrine signals, such as *Wnt* or *Fgfs* [45,46]. Post-transcriptional regulation is widely modulated by non-coding RNAs—mainly microRNAs and lncRNAs [47,48]. First evidence of requirement of microRNA function to proper cardiogenesis was contributed by Dicer conditional knockout murine models. Dicer mutant mice exhibits a double outlet right ventricle with a concurrent ventricular septal defect [49]. Furthermore, several microRNAs-mutant mouse models—*miR-1, miR-133, miR-17, miR-92* and *miR-138* [50,51,52,53,54]—have been developed to analyze their functional impact in early cardiogenesis modulating the proper commitment of cells to cardiac-specific muscle lineage and demonstrating a pivotal role of microRNAs on it. Furthermore, role of *miR-208a/b* and *miR-499* have been described as regulators on late cardiogenesis, required to correct expression of slow/fast muscle fibres genes and sarcomeric contractile function [55,56,57].

In this review we describe the impact of *miR-1/miR-133* cluster in heart development and its role in heart failure and pathological processes—cardiac fibrosis, cardiomyocytes hypertrophy, angiogenesis and inflammatory response—associated with it.

## 2. Genomic Organization and Transcriptional Regulation of *miR-1/miR-133* Cluster

**Figure 1:**
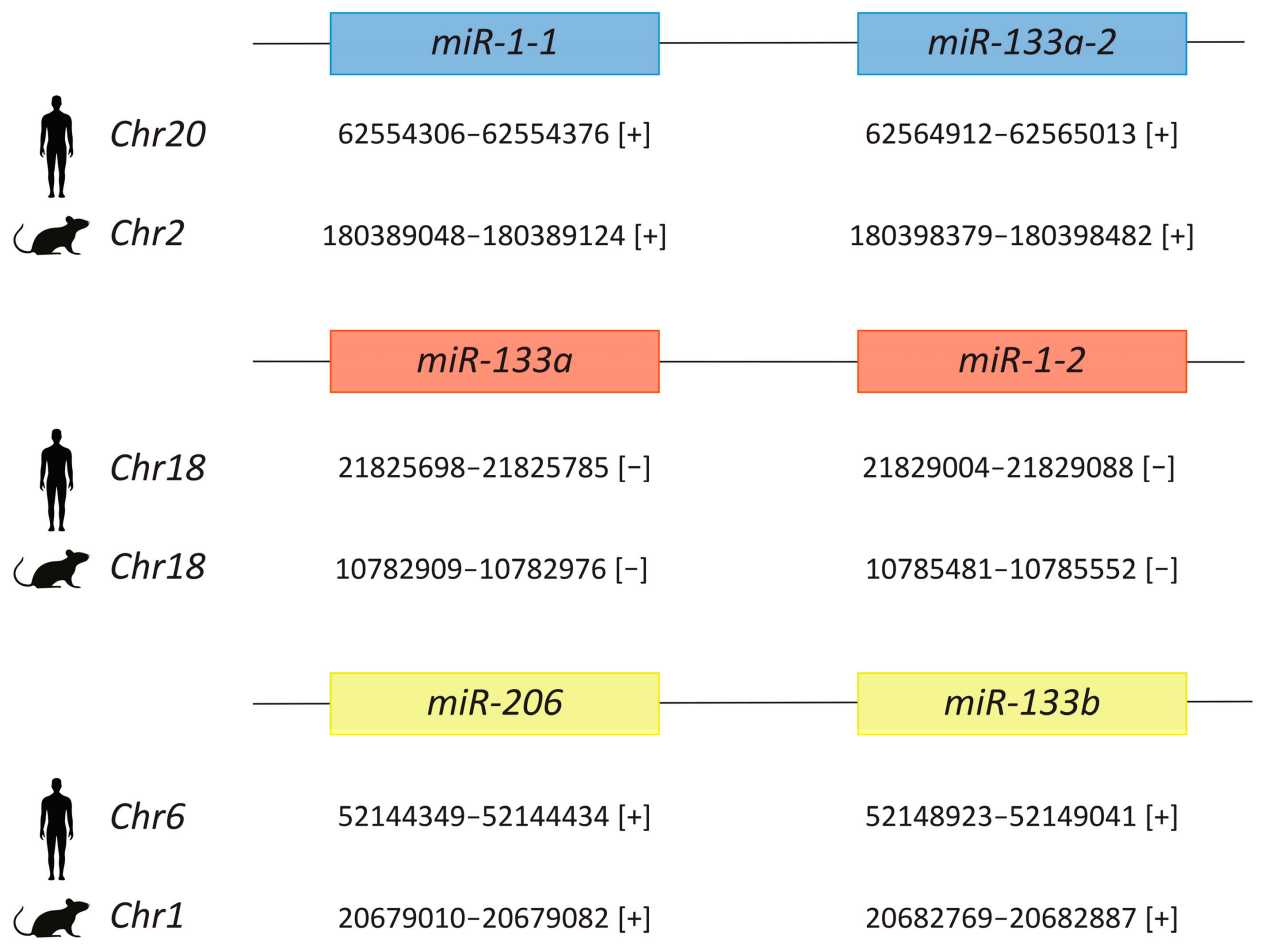
Genomic organization of *miR-1* and *miR-133*. Schematic representation of the genomic organization of *miR-1* and *miR-133* family members, highlighting their bicistronic clusters and their chromosomal locations.

These two microRNAs, *miR-1* and *miR-133*, are highly conserved between different species such as human, mouse, fish, chick, fly and worm. Both miRNAs, *miR-1* and *miR-133*, are derived from a bicistronic common precursor transcript, which is encoded by three different gene clusters, summarized in Figure 1: (I) *miR-1-1/miR-133a-2* gene cluster, located in chromosome 20 and 2—both in human and mouse species, respectively—in an intergenic region, encode for *miR-1-1* and *miR-133a-2* [50,58]. (II) *miR-1-2/miR-133a* gene cluster, located in chromosome 18 in human and mouse species, in an antisense orientation on intron 12 of the mindbomb ubiquitin-protein ligase E3 (Mib1), encode for *miR-1-2 and miR-133a*. (III) *miR-206/miR-133b* gene cluster, located in chromosome 6 and 1—both in human and mouse species, respectively—encode for *miR-206* and *miR-133b*. The mature forms of *miR-1-1/1-2* and *miR-133a/133a-2* derived from different gene clusters are identical, while *miR-206* differs of *miR-1-1/1-2* in four nucleotides, and *miR-133b* differs of miR-133a/133a-2 in only single nucleotide of 3’ end. Also, *miR-1-1/miR-133a-2* and *miR-1-2/miR-133a* clusters are highly expressed in both skeletal and cardiac muscle, whereas *miR-206/miR-133b* cluster expression is only detected in skeletal muscle during developmental and adult stages [51,59,60].

**Figure 2:**
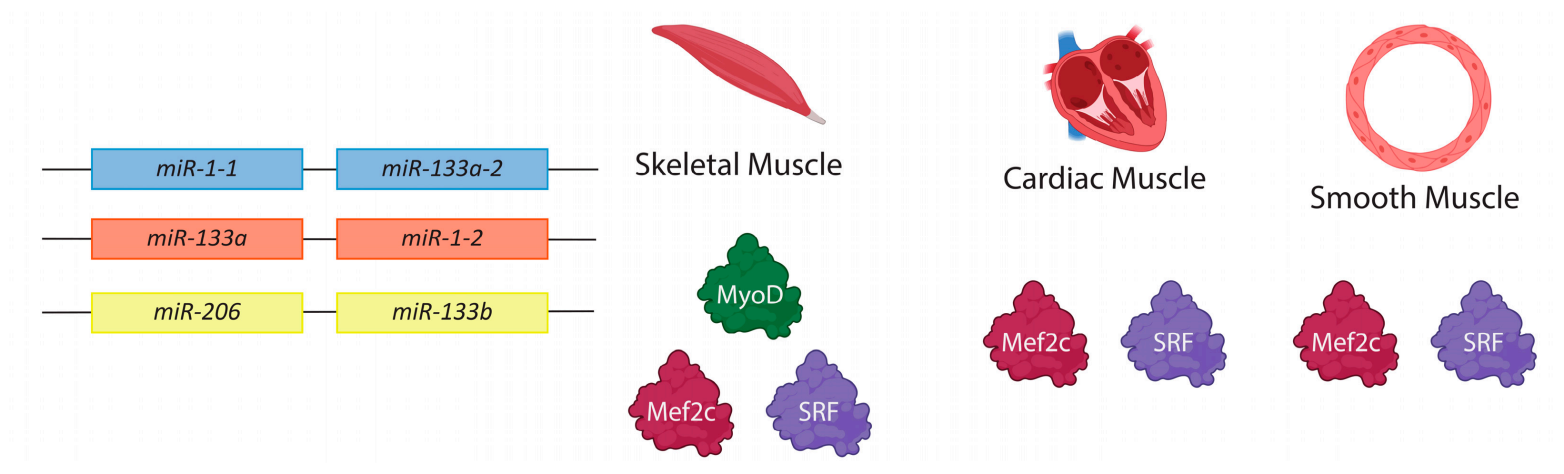
Transcriptional regulation of *miR-1* and *miR-133*. Overview of the transcriptional regulation of *miR-1* and *miR-133*, illustrating key transcription factors and their tissue-specific or cardiac domain-specific regulatory roles.

The transcription of both microRNAs in cardiac and skeletal muscles is addressed directly by several transcription factors (Figure 2), including myogenic differentiation factor (*MyoD*), serum response factor (*Srf*), and myocyte enhancer factor-2 (*Mef2*). *MyoD* orchestrates transcription of *miR-1* and *miR-133* in skeletal muscle exclusively, while *Srf* and *Mef2* regulate expression of them in cardiac, skeletal and smooth muscles. *Srf* recognizes and binds to specific enhancer sequence, which is widely conserved from flies to humans, located on 2.6 Kb and 0.6 KB upstream of *miR-1-1/miR-133a-2* and *miR-1-2/miR-133a* gene clusters, respectively. Curiously, binding of Srf to the enhancer sequences directs expression of *miR-1/miR-133* in ventricular chamber and outflow tract but not in atrial chambers. Although the upstream enhancers also contain MEF2-binding motifs, these sites are dispensable for their cardiac activity [50,51]. Unlike *Srf*, *Mef2* enhancer is active in both ventricular and atrial chambers and is located via an intragenic muscle-specific enhancer, within the *miR-1-2/miR-133a* gene cluster, but not on *miR-1-1/miR-133a-2*. Transgenic models have shown that both enhancers—upstream and intragenic—are functional from ED8.5 and their activation are required to proper early cardiogenesis [61].

Recently, non-canonical nuclear functions of microRNAs have been reported. Broadly, *miR-1* and *miR-133a* exert their function within the cytoplasm by canonical mRNA repression [62,63]. Nevertheless, it has been shown [26] that *miR-133a* modulates a transcriptional repression of de novo DNA methyltransferase 3B (*Dnmt3b*) in the nucleus of murine and human cardiomyocytes. Translocation of *miR-133a* to the nucleus is dependent on Wnt signalling repression and AGO2. Mechanistically, *miR-133* interacts with AGO2 in the cytoplasm, forming a miR-133a/AGO2 complex. Subsequently, *miR-133a*/AGO2 complex binds to karyopherin β family member importin 8 (IPO8), which in turn exerts the shuttling of the *miR-133a*/AGO2 complex from the cytosol to the nucleus. Once at nucleus, *miR-133a*/AGO2 complex mediates an epigenetic rearrangement within the Dnmt3b promoter and induces Dnmt3b to methylate its own promoter, establishing a *miR-133a*-mediated negative feedback loop. Functional assays demonstrated that *miR-133a*/AGO2 modulates a proper transcription of *Dnmt3b* in the early phases of cardiac differentiation of human pluripotent stem cells (hPSCs), contributing to correct proper differentiation and maturation of cardiomyocytes from these hPSCs. A corresponding nuclear function has not yet been demonstrated for *miR-1* in cardiac cells. Key questions are whether *miR*-1 enters the nucleus as an AGO2-containing complex, whether it recognizes promoter DNA, nascent RNA, or chromatin-associated lncRNAs, and whether any such interaction represses or activates cardiac genes. These possibilities could be tested by combining rigorous nuclear fractionation and single-molecule RNA imaging with AGO2 eCLIP or miRNA-centred chromatin isolation, followed by nascent RNA sequencing and chromatin profiling after nuclear-selective AGO2 or IPO8 perturbation. Seed-mutant and nuclear-localization rescue experiments would be required to distinguish direct nuclear activity from secondary cytoplasmic effects. Until such evidence is obtained, nuclear regulation by *miR-1* should be regarded as a testable mechanistic hypothesis rather than an established cardiac pathway [24,25,27].

Resolving the subcellular functions of *miR-1* and *miR-133* is therefore essential for understanding how the same bicistronic transcripts can coordinate post-transcriptional repression with possible transcriptional or epigenetic regulation during cardiogenesis and cardiovascular disease.

In sum, the genomic organization, enhancer-dependent transcriptional control, and emerging nuclear activity of the *miR-1/miR-133* clusters constitute a multilayered regulatory system that coordinates cardiac gene expression at the transcriptional, post-transcriptional and epigenetic levels.

## 3. Gene Expression Patterning of *miR-1* and *miR-133* During Cardiogenesis

**Figure 3:**
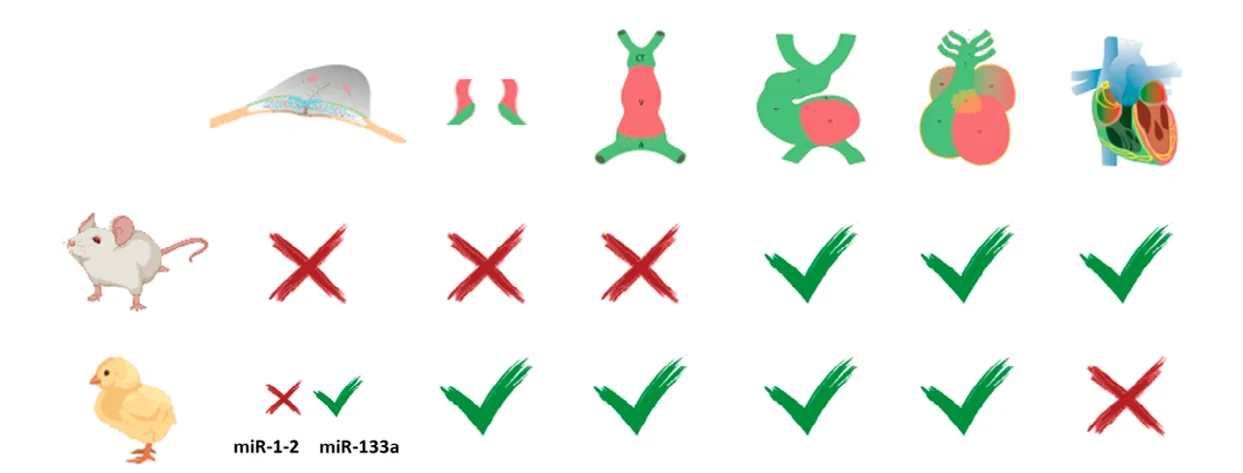
Expression patterning of *miR-1* and *miR-133a* during cardiogenesis. Illustrative patterning of *miR-1* and *miR-133a* gene expression throughout different stages of cardiogenesis, indicating their spatiotemporal presence during heart development. Note that, at earliest stages in chicken embryo, *miR-1-2* is not expressed. Red cross miRNAs not expressed, green tick miRNAs expressed.

In situ hybridization analysis in mouse and chicken embryos have revealed a broad expression of both *miR-1* and *miR-133* during cardiogenesis (Figure 3). In mouse embryo, both microRNAs are firstly detectable into the primitive cardiac tube (E8.5) and cardiac looping (E9.5), becoming robustly expressed from E10.5 to adult stage, both in ventricular and atrial myocardium (Figure 3). On the other hand, the expression of these microRNAs begins earlier in chicken embryo (Figure 3). Thus, *miR-1* and *miR-133a* expression is detectable from primitive endocardial tubes (HH8-), primitive cardiac tube (HH10), to the early formation of cardiac loop, where the intensity is decreasing in the anterior–posterior sense, disappearing in the inflow tract region and remaining in primitive ventricles [50,60,64,65,66]. Even more, *miR-133a* expression is observed in the chicken embryo from earlier stages [67], into the primitive streak precardiac cells (HH3) and first heart field (HH5).

Curiously, expression analysis in other species, such as *Xenopus* and zebrafish, have demonstrated that both *miR-1* and *miR-133a* are expressed in muscle progenitors, but not in the developing embryonic heart, suggesting that specific expression of both microRNAs in heart during cardiogenesis is exclusive of amniotes [68,69]. This apparent amniote–anamniote difference should be interpreted cautiously because low or transient cardiac expression may fall below the sensitivity of whole-mount in situ hybridization, and the probes used may not resolve individual paralogues. A plausible evolutionary explanation could be that the mature microRNA sequences and core cardiogenic transcription factors remained conserved while the cis-regulatory enhancers controlling cardiac expression diverged. Non-amniotes may therefore use different enhancer–factor combinations, expression windows, or partially redundant microRNAs to regulate analogous targets. Spatial small-RNA sequencing, locus-specific knock-in reporters, and comparative enhancer-accessibility assays will be required to distinguish true absence from low-level, stage-restricted expression. Thus, conservation of the mature microRNAs does not necessarily imply conservation of their embryonic cardiac cis-regulation.

Taken together, these findings reveal that *miR-1* and *miR-133* display evolutionarily conserved sequences but species-specific cardiac expression patterns, highlighting the importance of cis-regulatory divergence in shaping their spatiotemporal activity during cardiogenesis.

## 4. Functional Role of *miR-1* and *miR-133* in Cardiogenesis and Cardiomyocyte Differentiation

### 4.1. Role of miR-1 and miR-133 in Heart Development

**Figure 4:**
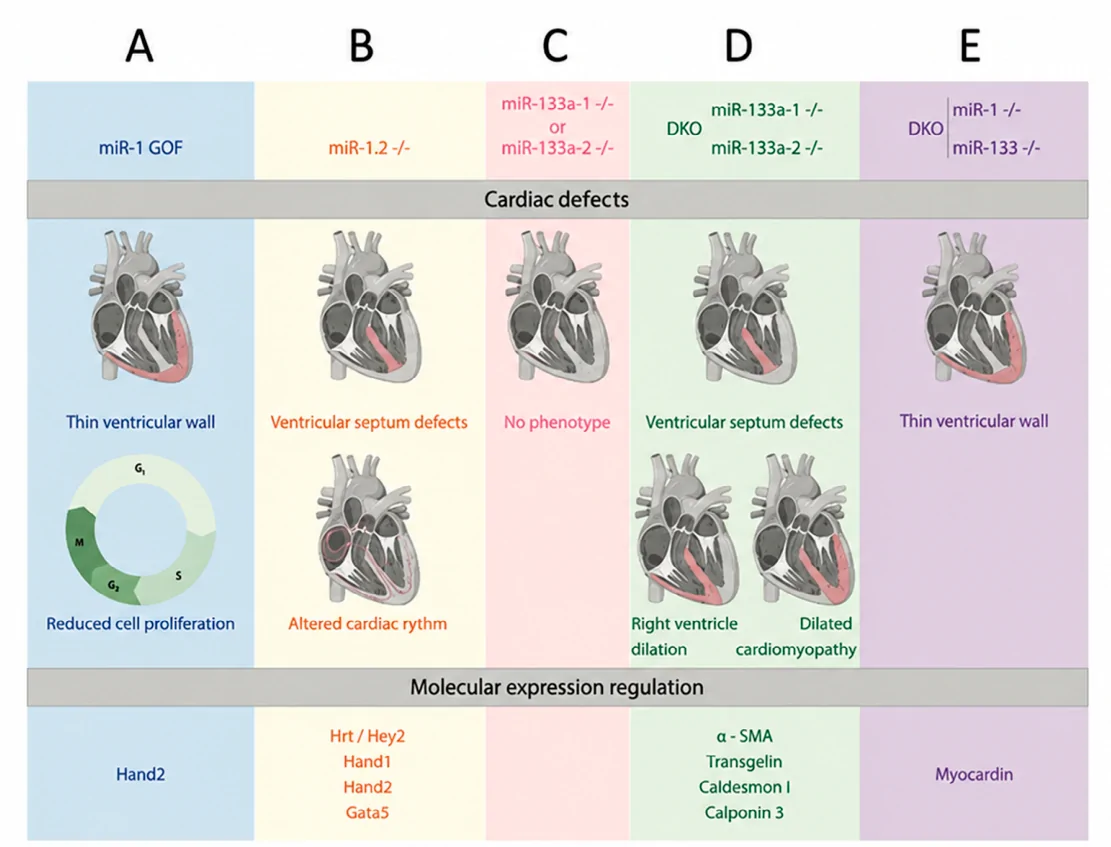
Functional roles of *miR-1* and *miR-133* in cardiogenesis. Critical functions of *miR-1* and *miR-133* in heart development, based on (**A**) gain- and (**B**–**E**) loss-of-function studies, highlighting primary cardiac defects and related key molecular targets. (**A**): Gain-of-function (GOF) of *miR-1*, which recognize *Hand2*, gives rise to thin-walled ventricles, reduced cell proliferation. (**B**): *miR-1-2* knockout mice present upregulation of specific genes, giving rise to ventricular septal defects and cardiac rhythm alterations. (**C**): Mice lacking *miR-133a-1* or *miR-133a-2* do not display any pathological phenotype. (**D**): Double mutants (DKO) *miR-133a-1/miR-133a-2* mice present upregulation of smooth-muscle gene programme, and display ventricular septal defects, dilated cardiomyopathy and heart failure. (**E**): DKO *miR-1/miR-133* mice present upregulation of *myocardin* and display thinning of ventricular walls.

Critical relevance of *miR-1* and *miR-133* in heart developmental have been addressed by different gain- and loss-of-function mutant murine models in vivo [50,51,60,70,71] as summarized in Figure 4. Overexpression of miR-1 under control of the β-MHC promoter resulted in E13.5 developmental arrest, due to impaired ventricular chamber formation and thus cardiac function (Figure 4A). Overexpression of *miR-1* gives rise to thin-walled ventricles formed by 3–5 cell layers, in contrast to control embryos, which presented 8–10 cell layers. Analysis of mitogenic activity demonstrated a significant decrease in the number of proliferating myocardial cells in *miR-1* transgenic mice, but no increase in apoptotic cells. Decreased pool of cardiomyocytes by *miR-1* upregulation resulted in failure of ventricular chamber expansion and therefore the inability to support blood flow through the embryo. Functional assays showed that the phenotype of *miR-1* transgenic mice is caused by downregulation of Hand2, a pivotal transcription factor required to correct ventricular myocyte expansion and cardiomyocyte differentiation. Mechanistically, *miR-1* recognizes and binds to Hand2, triggering Hand2 mRNA degradation and thus avoiding translation. As result, cardiomyocytes from *miR-1* transgenic mice display premature differentiation and early cell cycle withdrawal. Supporting the modulation of Hand2 by *miR-1*, deficient *Hand2* mice display E10.5 embryonic lethality due to right ventricular hypoplasia and decreased trabeculation in the left ventricle, similar to phenotype observed in overexpression *miR-1* mutant mouse [50].

On the other hand, *miR-1-2* knockout mice exhibit a less severe phenotype as compared to *miR-1* overexpression transgenic mice (Figure 4B). The deletion of *miR-1*-*2* produces an early lethality—between late embryogenesis and neonatal stage—in approximately 50% of the animals, but not in all of them, unlike what was observed in the overexpression model. This *miR-1-2* deficient mouse presents a spectrum of abnormalities, including ventricular septal defects (VSDs) in a subset that suffer early lethality, cardiac rhythm alterations in those that survive, and a striking myocyte cell-cycle abnormality that leads to ventricular hyperplasia of the heart with nuclear division persisting postnatally. Expression analysis of mutant hearts showed upregulation of pivotal cardiac genes related to VSDs phenotype, such as *Hrt2/Hey2, Hand1, Hand2 and Gata5*. Deletion of *miR-1-2* does not modulate expression of *miR-1-1*, demonstrating that appearance of phenotype associated with *miR-1-2* knockout mouse is exclusively caused by *miR-1-2* loss-of-function, suggesting that *miR-1-1* and *miR-1-2* exert their function in different pathways and/or on different targets [51,70].

Unlike *miR-1-1/miR-1-2*, mice lacking *miR-133a-1* or *miR-133a-2* are normal and do not display any pathological phenotype (Figure 4C). However, mice lacking both microRNAs (Figure 4D) resulted in embryonic lethal VSDs in approximately 50% of these double mutants (dKO), while surviving mutant mice develop dilated cardiomyopathy and heart failure. dKO hearts displayed a reduced compact free wall of the right ventricle and chamber dilatation. Furthermore, dKO hearts exhibit large VSDs near the apex and atrioventricular valve, generated by hypocellularity of the interventricular septum. Functional assays demonstrated that loss-of-function of both miRNAs results in ectopic expression of smooth-muscle gene programme, such as smooth muscle actin, transgelin, caldesmon 1 or calponin 3, which in turn positively modules differentiation of smooth muscle lineage in detriment of the cardiac muscle lineage. Secondly, loss-of-function of both microRNAs produces an aberrant cardiomyocyte proliferation rate by upregulation of several genes, such as *CyclinD1 and D2*. Conversely, gain-of-function of *miR-133a* in vivo reduces cardiomyocyte proliferation, leading to embryonic lethality due to thinning of ventricular walls and VSDs. Thus, the consequences of overexpression and loss-of-function demonstrate that *miR-133a* inhibits cardiomyocyte proliferation in the developing heart [60].

In addition to the abovementioned data, double mutants (dKO *miR-1/miR-133*) display severe cardiac defects (Figure 4E), including thinning of ventricular walls, owing to a striking reduction in the cardiomyocte proliferation rate, resulting in an early lethality, around E11.5 [71]. Furthermore, the detailed analysis of dKO cardiomyocytes revealed an immature differentiation state, showing a mixed smooth-muscle-cardiac muscle phenotype. In this context, it is observed an upregulation of *myocardin*, a pivotal modulator of smooth muscle gene programme, demonstrating that *miR-1* and *miR-133* are required for proper specification of cardiomyocite lineage, and suggesting that cardiac defects observed in dKO are caused by an early arrest of cardiomyocyte differentiation.

Further evidence on the functional role of *miR-1* and *miR-133* have also been reported in avian cardiogenesis [65,66]. In this sense, we have demonstrated that both *miR-1* and *miR-133a* are required for proper differentiation of posterior cardiac segment and its derivatives. The complementary expression of *miR-1* and *miR-133a* modulates the activation or repression of retinoic acid (RA) signalling during early cardiogenesis, respectively. Thus, *miR-133a* represses *RhoA* and *Cdc42* expression, resulting in indirectly downregulation of *Raldh2* and disruption of RA signalling in cardiac structures derived from posterior cardiac segment. Functional assays demonstrated that the downregulation of *miR-133a* on posterior cardiac tube results in increased expression of *Raldh2* and therefore enhanced RA signalling. As consequence, primitive atrium and sinus venosus display a reduced cardiomyocyte proliferation and severe hypoplasia [65]. On the other hand, *miR-1* activates the expression of *CrabpII* and *Rarβ*, promoting RA signalling and, therefore, increasing expression of *Amhc1*, *Gata4* and *Tbx5*, while *Mef2c* is repressed [66]. Contrary to *miR-133a* overexpression, *miR-1* upregulation is translated into increased cardiomyocyte proliferation and severe hyperplasia of primitive atrium and sinus venosus. All these data support that both microRNAs play antagonistic roles defining the early limits of the posterior cardiac region and its derivatives, by modulating the activation of RA signalling and thereafter the proliferation and maturation of cardiomyocytes.

Thus, these gain- and loss-of-function studies demonstrate that *miR-1* and *miR-133* are dose-sensitive and context-dependent regulators of cardiogenesis. Excessive *miR-1* restricts ventricular growth through *Hand2* repression, whereas the incomplete compensation of *miR-1-2* loss by *miR-1-1* highlights the importance of locus-specific dosage and spatiotemporal regulation. Similarly, *miR-133a* limits cardiomyocyte proliferation and prevents inappropriate activation of the smooth-muscle gene programme, while the severe phenotype caused by the combined loss of both clusters demonstrates their synergistic requirement for cardiomyocyte specification and maturation. Conversely, avian studies reveal that *miR-1* and *miR-133a* can act antagonistically by fine-tuning RA signalling, thereby controlling cardiomyocyte proliferation and posterior cardiac patterning. Overall, their functional relationship depends on dosage, developmental stage, cellular context and the biological process examined.

### 4.2. Functional Role of miR-1 and miR-133 in Mesoderm and Cardiomyocyte Differentiation

**Figure 5:**
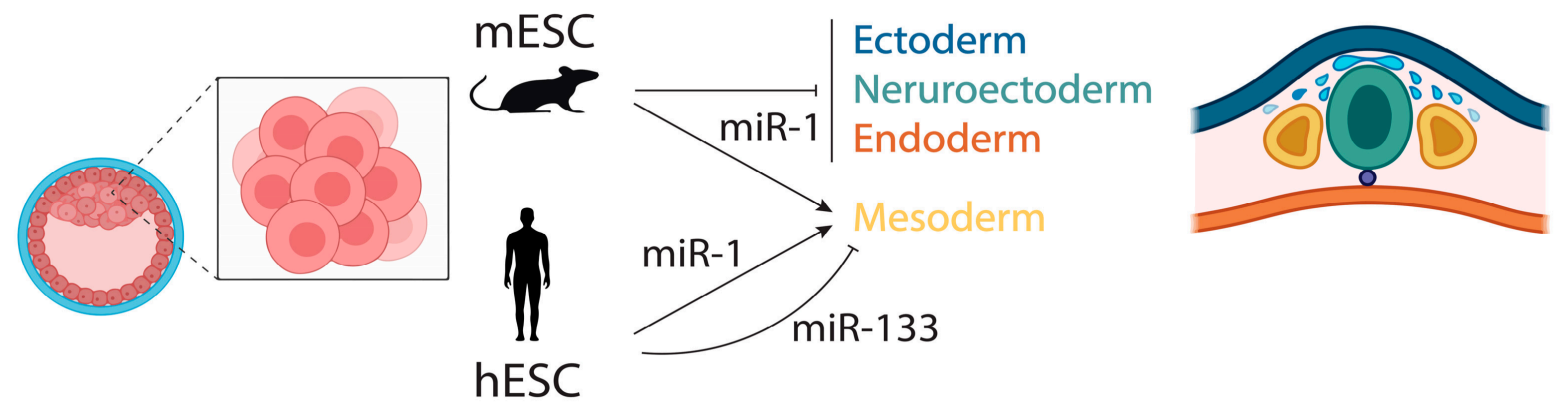
Roles of *miR-1* and *miR-133* in cardiac differentiation. Functional involvement of *miR-1* and *miR-133* in mesoderm and cardiomyocyte differentiation from stem cells in mice and humans. Blue: ectoderm. Green: neuroectoderm. Orange: endoderm. Yellow: mesoderm.

Several in vitro studies have demonstrated that *miR-1* and *miR-133* are required for proper differentiation of mesodermal precursors from embryonic stem cells (ESCs). Upregulation of *miR-1* in ESCs promotes mesodermal lineage by increasing the expression levels of *Nkx2*.5 and *Cdk9*, while blocking differentiation towards endodermal, neuroectodermal and ectodermal lineages (Figure 5). Similarly*, miR-133* recognizes and targets *Egfr*, inhibiting ectodermal differentiation [59]. Additionally, the co-localization of *miR-1* with myosin-positive cells in embryonic bodies suggests that expression of *miR-1* is restricted to myogenic precursors. Furthermore, upregulation of *Srf* in ESCs increases *miR-1* expression, which in turn represses Notch ligand Delta Like-1 (Dkl1), inhibiting differentiation towards non-muscle lineage [72,73].

In line with these findings, Lu et al., 2013 [74], reported increased levels of *miR-1* in human cardiomyocytes, but not in endothelial cells (ECs) or smooth muscle cells derived from ESC multipotent cardiovascular progenitors (MCPs). Upregulation of *miR-1* in MCPs blocks Wnt and Fgf signalling by targeting Frizzled-7 (Fzd7) and fibroblast growth factor receptor substrate 2 (Frs2), respectively, directing differentiation towards cardiac muscle cells [74]. Unlike *miR-1*, *miR-133* blocks differentiation from ESCs to cardiomyocyte precursors by downregulating several cardiac markers such as *Nkx2*.5, *Gata4* and *Mef2c* [71].

On the other hand, microinjection of *miR-133a* on precardiac areas in chicken embryos during early cardiogenesis resulted in downregulation of *Fgf8* and upregulation of *Bmp2* promoting cardiac induction of mesoderm derivatives [67].

Taken together, these studies indicate that *miR-1* and *miR-133* exert complementary but stage-dependent functions during mesodermal and cardiac differentiation. *miR-1* consistently promotes commitment towards the myogenic and cardiomyocyte lineages by activating cardiac transcriptional programmes and repressing signalling pathways associated with alternative cell fates. By contrast, *miR-133* suppresses ectodermal differentiation and can promote early cardiac mesoderm induction through modulation of *Fgf8/Bmp2* signalling, while limiting the subsequent transition of embryonic stem cells into cardiomyocyte precursors. Thus, the effects of *miR-1* and *miR-133* depend on the developmental stage and cellular context, enabling them to fine-tune the balance between lineage specification, progenitor maintenance, and terminal cardiac differentiation.

### 4.3. Role of miR-1 and miR-133 in Cellular Reprogramming

**Figure 6:**
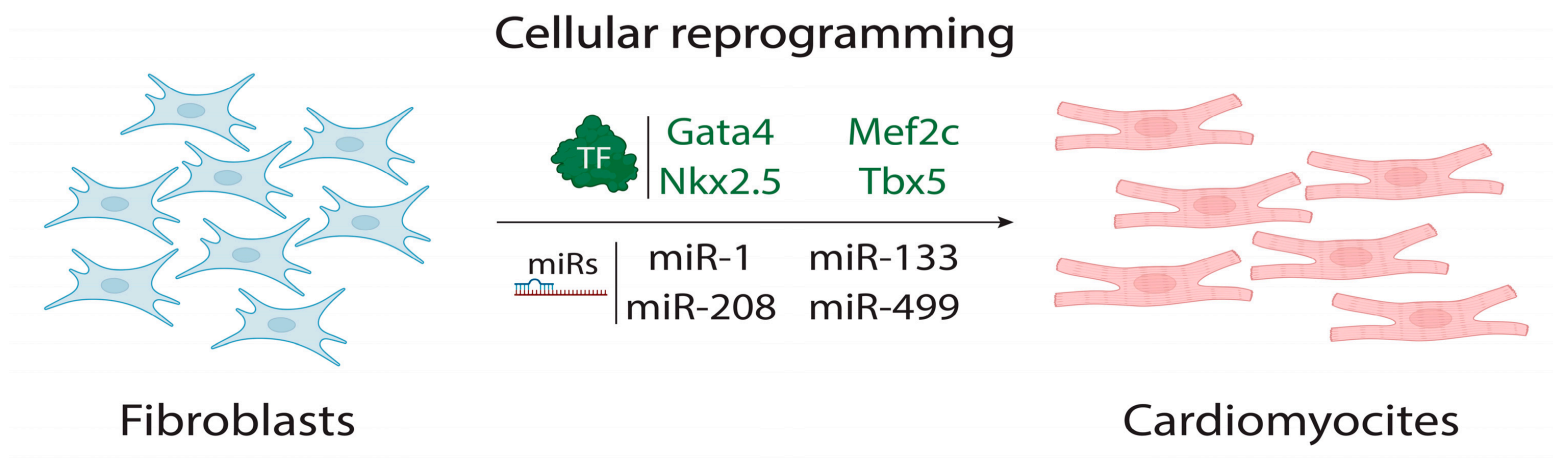
Roles of *miR-1 and miR-133* in cellular reprogramming. Role of *miR-1* and *miR-133* in the direct cellular reprogramming of fibroblasts into induced cardiomyocyte-like cells (iCMs).

The potential role of *miR-1* and *miR-133* as differentiation modulators of cardiomyocytes is reflected by their capacity to mediate cellular reprogramming of fibroblasts into cardiomyocyte-like cells (iCMs) [75,76,77,78,79]. Reprogramming resident fibroblasts into new functional cardiomyocytes have been described as potential approach of regenerative therapies against cardiomyocyte injuries such as myocardial infarction (MI) and other cardiovascular remodelling diseases [80,81]. Direct cardiac reprogramming of resident fibroblast (Figure 6) has been reached both via transcription factors—*Gata4*, *Mef2c*, *Nkx2.5*, *Tbx5*—or microRNAs [75,82]. It has been demonstrated [75] that administration of *miR-1* and *miR-133*, both in vivo and in vitro, accompanied with miR-208 and miR-499 (miR-Combo) mediated direct conversion of fibroblasts into cardiomyocytes. As a result of miR-Combo administration, resident fibroblasts switch fibroblastic pattern expression to early cardiogenic expression pattern, upregulating genes such as *Nkx2-5*, *Gata4*, *Tbx5*, *Islet1* and *Mef2c*, as well as late cardiogenic markers, such as Connexin 43 and cardiac troponin I. Moreover, cardiomyocyte derived from resident fibroblasts exhibit a contractile phenotype characterized by calcium depolarization. The in vivo approach has shown that administration of miR-Combo, by intrapericardial injection after MI, results in a major cardiac function and a lower loss of cardiomyocytes in the infarcted myocardium. Furthermore, functional assays have shown that expression of cardiogenic gene program in resident fibroblasts from miR-Combo administration requires an intensive remodelling of the epigenetic environment mediated by H3K27 demethylation [76].

Curiously, administration of transcription factors *Gata4*, *Mef2c* and *Tbx5* are effective to reprogramming fibroblasts to iCMS in mice but not in human or pig cells. In contrast to the transcription factor approach, miR-Combo is effective in several mammalian models such as mice, pigs or dogs, suggesting a potential effective reprogramming function in human, emerging as prospective therapeutic use [83,84].

Overall, these studies identify *miR-1* and *miR-133* as key components of miR-Combo-mediated cardiac reprogramming, promoting the conversion of resident fibroblasts into cardiomyocyte-like cells through activation of cardiogenic transcriptional programmes and epigenetic remodelling. The resulting cells acquire cardiac markers, calcium-handling properties, and contractile activity, while in vivo administration improves cardiac function and limits cardiomyocyte loss following myocardial infarction. Moreover, the efficacy of miR-Combo in several mammalian models, in contrast to the limited cross-species activity of transcription-factor-based approaches, supports its translational potential. Nevertheless, reprogramming efficiency, cellular maturity, targeted delivery, and long-term safety must be rigorously evaluated before clinical application.

## 5. Role of *miR-1* and *miR-133* in Adult Cardiac Homeostasis and Heart Failure

### 5.1. Myocardial Infarction and Cardiac Hypertrophy

Heart failure (HF) is a progressive syndrome associated with high morbidity and mortality, impaired quality of life, and high healthcare costs. Globally, more than 55 million people live with HF, and prevalence is steadily rising [85]. HF usually involves complex clinical profiles, frequently found at late stages of numerous heart diseases, originating from functional and structural ventricular impairment, and resulting in symptomatic left/right ventricle (LV/RV) dysfunction [86,87,88]. As a direct consequence of HF, a large number of cardiomyocytes undergo apoptotic processes, leading to irreversible myocardial damage, subsequently triggering cardiac dysfunction. Several pathophysiological processes may precede HF, including ventricular and atrial remodelling accompanied by cardiomyocyte hypertrophy, interstitial fibrosis, exacerbated inflammatory response and impaired arrhythmogenesis, among others [89,90,91].

Several studies have broadly described the regulation of genetic pathways involved in the development and progression of pathological processes responsible for HF. In particular, non-coding RNAs have been shown to play a pivotal role both as protective and harmful modulators of cardiovascular diseases and derived pathological processes, by means of post-transcriptional and transcriptional regulation. Specifically, several microRNAs and lncRNAs have been considered as therapeutic tools in HF development, prognosis and recovery. Therefore, it is of vital importance to elucidate the role of microRNAs and lncRNAs in those molecular pathways involved in HF and its associated pathological processes [92,93,94].

**Figure 7:**
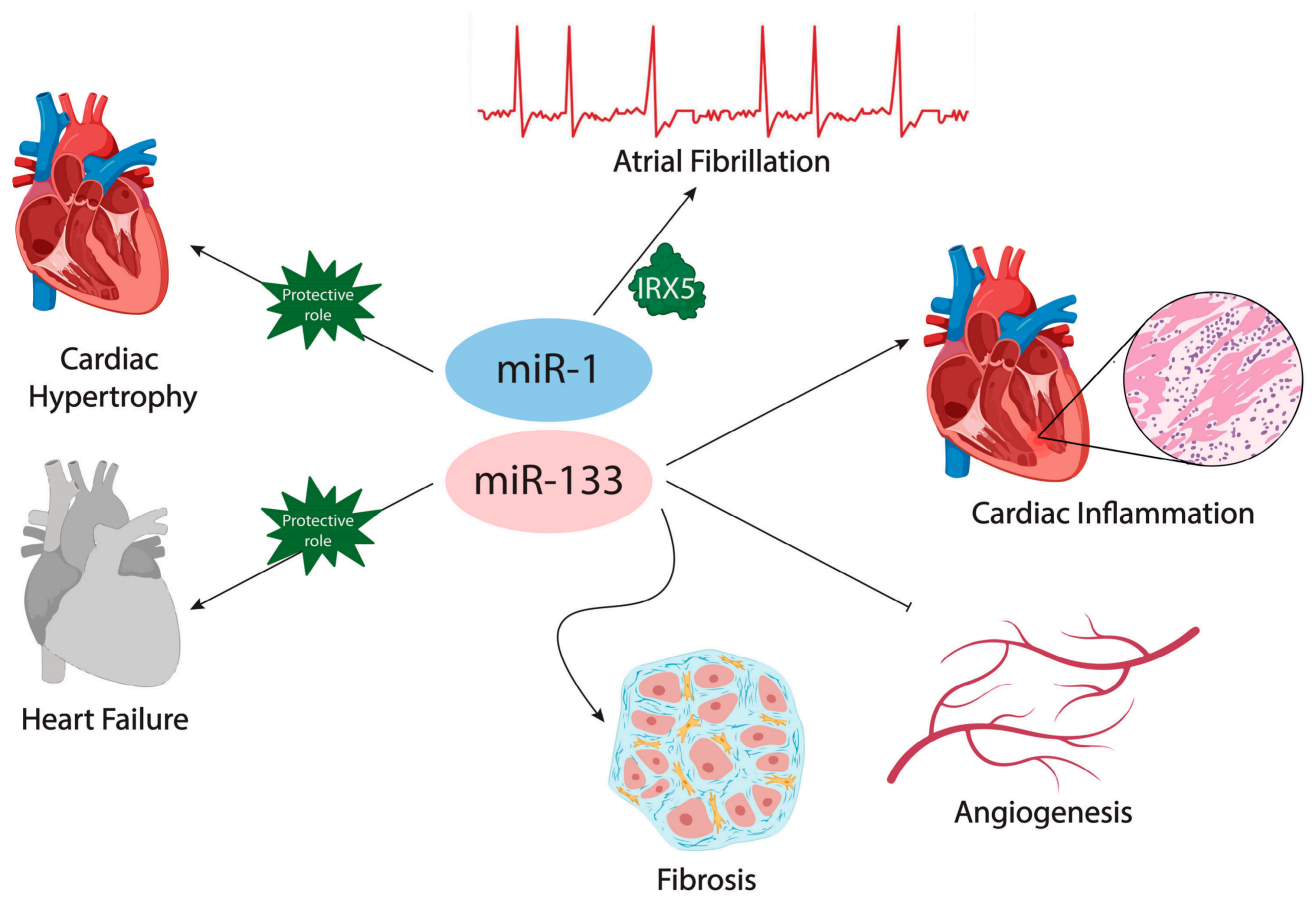
Roles of *miR-1* and *miR-133* in cardiac pathologies. Functional implications of *miR-1* and *miR-133* in major cardiac pathologies, depicting their protective or modulatory roles in these conditions.

A large number of studies have reported that *miR-1* and *miR-133* play a protective role against HF (Figure 7) and associated pathological processes [95,96,97,98,99,100,101]. The expression analysis of these microRNAs have shown a marked downregulation in infarcted cardiac tissues, observed in humans, rats and mice. Noticeably, in human post-mortem samples, a comprehensive analysis has shown that *miR-133* is repressed both in the infarcted region and the surrounding infarcted area, while *miR-1* is only repressed in the former region, suggesting a specific functional role of each microRNA in the crosstalk between necrotic damage cardiomyocytes and surrounding cells [102,103].

Several studies carried out on cardiomyocytes isolated from different cardiac hypertrophy in vivo models (mediated by thyroid hormones, transverse aortic constriction and isoproterenol and angiotensin II administration) present a marked downregulation of *miR-1* and *miR-133*, suggesting they are negatively modulated by pro-hypertrophy signalling pathways [99,100,104,105,106]. Moreover, both in vitro and in vivo functional assays have demonstrated that *miR-1* and *miR-133* prevent cardiac hypertrophy response chiefly by repressing calcium signalling pathway (Figure 7). Both microRNAs modulate distinct pivotal genes involved in pro-hypertrophic calcium signalling. In particular, in human cardiomyocytes, *miR-133a* gain-of-function represses translation of Calcineurin (*CaN*), a calcium-dependent serine/threonine protein phosphatase, which is pivotal upstream modulator of cardiac hypertrophy. Also, *CaN* modulates the translocation of Nuclear Factor of Activated T-Cells 3 (*Nfact3*) from the cytoplasm to the nucleus, inducing the expression of MAPK signalling dependent genes, required for cardiomyocyte hypertrophic response development [107]. On the other hand, *miR-1* is capable of binding to *Nfact3* mRNA, repressing its translation, whereas *miR-133a* recognizes the mRNAs of protein kinase C (*Pkc*) and *Nfact4*, which in turn promotes *Erk1/Erk2* and c-Jun expression [96,108]. Furthermore, *miR-1* represses translation of mitochondrial calcium uniporter (*MCU*), the pore-forming subunit of the mitochondrial Ca^2+^ uniporter complex (*MCUC*) [109]. *MCU* exerts an essential role in cardiomyocytes stress adaptation by controlling Ca^2+^ uptake in the mitochondria and its expression is upregulated in hypertrophied human cardiomyocytes [110]. Therefore, *miR-1* not only modulates the hypertrophic response by repressing pro-hypertrophic transcription factor, *Nfact3,* but also regulates the uptake and release of mitochondrial Ca^2+^, which is required to maintain the proper Ca^2+^ homeostasis and cardiac function [111]. Curiously, *miR-133a* partially represses translation of angiotensin II receptor subtype 1 (At1r) blocking the pro-hypertrophic cascade mediated by AngII. Inhibition of *Atr1* by *miR-133a* is translated into *Serca2* mRNA and protein reduction, suggesting that *miR-133a* modulates Ca^2+^ homeostasis of sarcoplasmic/endoplasmic reticulum in angiotensin II-induced cardiac hypertrophy. Hence, *miR-1* represses translation of signalling Nuclear Factor of Activated T-Cells cytoplasm 3 (*Nfact3*) and uptake/release mitochondrial Ca^2+^ whereas miR-133 inhibits upstream genes of hypertrophy pathways and the delivery Ca^2+^ from sarcoplasmic reticulum, displaying a complex and efficient inhibitory system of hypertrophic stimuli induced by calcium signals.

Additionally, *miR-1* represses cardiac hypertrophy by modulation of other pro-hypertrophic signals such as cytoskeleton remodelling or pro-hypertrophy epigenetic genome. It has been demonstrated [112] that upregulation of Twinfilin-1 (*Tw1*) on rat cardiomyocytes is translated in cardiac hypertrophy development. *Tw1* is a pivotal modulator of cytoskeleton dynamics and remodelling by binding actin monomers with high affinity and preventing their association with actin-filament barbed ends [113]. *Tw1* expression is upregulated by hypertrophy stimuli, displaying an aversely miR-1 expression. Curiously, *miR-1* is capable of binding to *Tw1* mRNA and trigger degradation. Functional assays have demonstrated that upregulation of miR-1 represses *Tw1* translation, avoiding cardiac hypertrophic response. Furthermore, it is known [104] that *miR-1* modulates the arrangement of epigenetic environment required for induction of cardiac hypertrophy by repressing histone deacetylase 4 (*Hdac4*).

These data support that upregulation of *miR-1* and/or *miR-133a* and, subsequently, the degradation of several effectors above described, ameliorates the cardiac hypertrophy response. Taken together, these findings indicate that *miR-1* and *miR-133a* act as complementary protective regulators against heart failure-associated myocardial remodelling. Their downregulation in infarcted and hypertrophic myocardium facilitates the activation of pathological pathways, whereas restoration of their expression restrains cardiomyocyte hypertrophy by targeting distinct but convergent components of calcium-dependent signalling. In addition, *miR-1* inhibits cytoskeletal and epigenetic remodelling through repression of *Tw1* and *Hdac4*, respectively. Thus, *miR-1* and *miR-133a* constitute a coordinated anti-hypertrophic regulatory network whose therapeutic modulation may help preserve calcium homeostasis, cardiomyocyte structure, and cardiac function.

### 5.2. Cardiac Fibrosis

Fibrotic environment in the heart is characterized by myocardial remodelling and accumulation of extracellular matrix (ECM). Cardiac-activated myofibroblasts acquire a limited contractile capacity and secrete fibrillar collagen and several ECM proteins to the extracellular environment. Their expansion following myocardial injury is primarily driven through activation of resident interstitial cell populations [114,115]. Several other cell types, including cardiomyocytes, endothelial cells, pericytes, macrophages, lymphocytes and mast cells may contribute to the fibrotic process, by producing proteases that participate in matrix metabolism, by secreting fibrogenic mediators and matricellular proteins, or by exerting contact-dependent actions on the fibroblasts phenotype [116,117].

Several in vitro and in vivo approaches have described that *miR-133a* (Figure 7), but not miR-1a, negatively modulates collagen synthesis and activation of profibrotic gene expression programme by cardiac fibroblasts. In transgenic mouse, *miR-133a* overexpression displays a severe blockage of cardiac fibrosis after myocardium injury by repressing *Erk1/2-Smad2* phosphorylation. On the other hand, a modulation of *miR-133a* and *SRF* gene has been involved in a fibrotic context. Note that *Srf* and its co-activator myocardin-related transcription factor A (Mrtf-A) represents one essential mechanism regulating myofibroblast activation after injury, where increased *Mrtf-A/Srf* signalling has been shown to activate cardiac fibroblasts to myofibroblasts. Srf over-expressing transgenic mouse exhibits a drastic *miR-133a* downregulation. Such repressed *miR-133a* expression is accompanied by increased cardiac fibrosis and alteration of cardiac gene expression program leading to upregulation of several factors involved in fibrotic response, including fibronectin, procollagen type 1α1, 3α1 and connective tissue growth factor (Ctgf). Curiously, Ctgf is repressed by *miR-133a*, by recognizing Ctgf 3’UTR and triggering its mRNA degradation [118,119]. Furthermore, expression of *miR-133a* is downregulated in a diabetes-induced cardiac fibrosis mouse model. Functional assays in this context demonstrated that *miR-133a* downregulates *Tgfβ* translation and therefore blocks the activation of profibrotic *Tgfβ* dependent effectors [120].

In sum, these findings identify *miR-133a* as a central negative regulator of cardiac fibrosis. By inhibiting ERK1/2–SMAD2 signalling and directly repressing profibrotic mediators such as CTGF and TGF-β, *miR-133a* limits myofibroblast activation, collagen synthesis, and extracellular-matrix deposition. Its reciprocal relationship with the MRTF-A/SRF pathway further suggests that *miR-133a* participates in a regulatory feedback mechanism controlling fibroblast activation following myocardial injury. Therefore, restoring *miR-133a* activity may represent a promising strategy for attenuating pathological cardiac fibrosis, whereas an equivalent antifibrotic role for *miR-1* remains insufficiently established.

### 5.3. Angiogenesis

Cardiomyocyte hypertrophy is accompanied by angiogenesis that is required to expand the coronary vasculature and warrants the proper supply of oxygen and nutrients to myocardium (Figure 7). In physiological hypertrophy, the heart preserves its oxygen supply by matching the proportional increases in cardiomyocyte size and the extent of angiogenesis by producing vascular endothelial growth factors, which modulate the development of new and larger blood vessels [121,122,123]. Aversely, in pathological states, cardiomyocyte hypertrophy is associated with an inhibition of the angiogenesis process and subsequently results in early development of heart failure [124,125].

Several authors [126,127,128] have demonstrated a negative modulation of angiogenesis by *miR-133a*, whereas the role of *miR-1* have yet to be addressed. In vitro approaches have shown that upregulation of *miR-133a* in endothelial cells (ECs) is translated into the reduction of proliferation rate, cell viability and cellular migration. Functional assays have shown that *miR-133a* recognized two pivotal pro-angiogenic factors, such as *Vgrf2* and *Fgfr1* mRNAs, triggering the degradation of both and impairing proper angiogenesis. Also, it has been demonstrated that *miR-133a* is required to maintain the quiescence phenotype of vascular smooth muscle cells (VSMCs); thus, in response to vascular injury, *miR-133a* expression is downregulated in VSMCs, both in vitro and in vivo, leading to increased cell proliferation rate and activation of two pivotal modulators of the smooth gene programme, e.g., *Sp1* and *Srf* [129].

Taken together, *miR-133a* exerts context-dependent vascular effects by inhibiting endothelial angiogenesis through *Vegfr2* and *Fgfr1* repression while maintaining vascular smooth-muscle-cell quiescence. This cell-specific duality must be considered when evaluating its therapeutic potential, whereas the vascular role of miR-1 remains unclear.

### 5.4. Inflammation

Inflammation plays a central role in the development of heart failure, especially in heart failure with preserved ejection fraction (HFpEF), as well as in several inflammatory cardiomyophaties. Furthermore, the inflammatory response enables the induction of regenerative processes following acute myocardial injury. The cellular response to myocardial ischemia can be categorized into different phases: the acute inflammatory phase, the healing phase, and a phase of chronic inflammation [130,131].

The role of *miR-1* and *miR-133* have been poorly explored in this context. Expression analysis of *miR-133a* in endo-myocardial biopsies from patients with inflammatory cardiomyophaty have shown that *miR-133a* upregulation is accompanied by increased myocardial inflammation and macrophage infiltration, suggesting that miR-133a exerts a harmful modulation in inflammatory response (Figure 7). However, additional studies are required to better understand the role of *miR-133a* in this process [132,133]. Recently, *miR-1* has been proposed as a promising therapeutic target for chronic heart failure, offering a novel approach to modulate cardiac function and inflammation, by suppression of *Tnf-α* and *Il-6* secretion, directly targeting hyperpolarization-activated cyclic nucleotide-gated channels 2 and 4 (*Hcn2/Hcn4*) axis, as analyzed in vitro [134].

Thus, current evidence suggests context-dependent inflammatory roles for *miR-1* and *miR-133a*: elevated *miR-133a* is associated with myocardial inflammation and macrophage infiltration, whereas *miR-1* may suppress *Tnf-α* and *Il-6* production through the *Hcn2/Hcn4* axis. Nevertheless, these limited findings require further mechanistic and clinical validation.

### 5.5. Arrhythmogenesis

It has been demonstrated [50] that *miR-1-2* deficient mouse displayed severe arrhythmias by impairing *Irx5* expression, a gene responsible for the establishment of the correct cardiac ventricular repolarization gradient (Figure 7). Increased *Irx5* expression represses transcription of transient outward potassium channel Kv4.2 by recruiting *mBop*, a cardiac transcription factor involved in cardiac conduction system development [50,135,136]. Furthermore, lower expression of *miR-1-2* has been reported in left atrium from persistent atrial fibrillation (AF) patients. Functional assays demonstrated that *miR-1-2* downregulation increases *Kcnj2* mRNA levels, which encodes the inwardly rectifying current channel, *Kir2.1*. As result of *Kcnj2* upregulation, cardiomyocytes exhibit an action potential duration shortening and increased K+ intracellular concentration promoting the atrial electrical re-entry and AF maintenance [137].

Interestingly, *miR-1-2* gain-of-function triggers a plethora of ventricular arrhythmogenic phenotypes. Gain-of-function of *miR-1-2*, both on rat ischemic model and ventricular tissue from explanted human hearts, promoted downregulation of *GJA3*, which encodes *Cx43*, and *Kcnj2*. Lower expression of *Cx43* slowed electrical conduction, while *Kir2.1* reduction attenuated *IK1* and prolonged ventricular repolarization, resulting in a widened QRS complex and a prolonged QT interval, leading to increased incidence of premature ventricular beats and ventricular tachyarrhythmia [138]. Noticeably, upregulation of *miR-1-2* modulates Ca^2+^ handling in rat and mouse ventricular cardiomyocytes. In this line, *miR-1-2* recognizes and binds to phosphatase PP2A regulatory subunit *B56α*, triggering mRNA degradation and avoiding its translation. This subunit is required by PP2A to target specialized subcellular domains. Reduced expression of *B56α* is translated in the hyperphosphorylation of ryanodine receptors, leading to increased functional activity of RyR2 channels, increasing the amplitude of the inward Ca^2+^ current, and enhancing frequency of spontaneous Ca^2+^ sparks, while reducing the sarcoplasmic reticulum Ca^2+^ content [111]. Increased resting Ca^2+^ is partially caused by repression of *Stxt6*, which is responsible for mediating intracellular transport of different ion channels, such as L-type calcium channels, toward organelle and cellular membrane. Also, it is known [139] that *miR-1-2* triggers *Stxt6* mRNA degradation, resulting in impaired intracellular traffic of ion channels and therefore generating a arrhythmogenic substrate. Moreover, *miR-1-2* overexpression in vivo caused atrioventricular block in mice by reducing systolic Ca^2+^ current and increasing the resting Ca^2+^, thus enhancing diastolic Ca^2+^ current in ventricular myocytes [140]. Finally, rescued assays have shown that administration of LNA-modified anti-miR-1 is sufficient to restore atrioventricular blockage in vivo [141]. Given the potential role of *miR-1* in cardiac conduction system, it has been proposed as a reliable circulating biomarker for AF [142].

The impact of *miR-133a* on cardiac electrophysiology diseases has also been explored in AF, and expression analysis in several samples from AF patients have demonstrated a marked *miR-133a* downregulation [143,144]. Previous studies [145] showed that *miR-133a* expression is drastically reduced in loss-of-function mutation of the zinc finger homeobox 3 gene (*Zfhx3*), being associated with increased risk of AF. Moreover, functional assays showed that *miR-133a/b* upregulation is able to reverse the atrial arrhythmia characteristic of *Zfhx3* knockdown. These data suggest a potential role of *miR-133* as a protective molecule against AF disease.

Finally, in vivo *miR-133a* gain-of-function experiments in rats [146] cause no changes in the heart pacemaker and basal heart rate, but substantially alter electrical activity in the working atrial and ventricular myocardium, inducing a QT-interval prolongation. Also, the functional assay showed that *miR-133a* upregulation induced repolarization abnormalities in the atrial working myocardium by increasing the L-type calcium current (I_Ca,L_), while increasing both I_Ca,L_ and I_to_ in ventricular cardiomyocytes. Additionally, luciferase analysis revealed that *miR-133a* targets both protein phosphatase 2 (*Ppp2ca/b)* and potassium voltage-gated channel-interacting protein 2 (*Kcnip2*) mRNAs, encoding Kchip2 (Kv Channel-Interacting Protein 2), a Kv4.3 potassium channel regulatory protein; these data suggest that electrical alterations are caused by downregulation of both genes.

Taken together, these findings demonstrate that *miR-1* and *miR-133a* are dose-dependent regulators of cardiac electrical activity whose deficiency and excessive expression can both promote arrhythmogenesis. Reduced *miR-1* disrupts repolarization gradients and favours atrial fibrillation through *Irx5-* and *Kcnj2*-dependent mechanisms, whereas its overexpression impairs electrical conduction and calcium handling by regulating *Cx43, Kir2.1, Pp2a, Ryr2, and Stx6.* Similarly, restoration of *miR-133a* may protect against atrial fibrillation, although excessive expression can alter calcium and potassium currents and prolong the QT interval. Therefore, therapeutic or biomarker applications of these microRNAs must consider disease type, cardiac compartment, dosage, and electrophysiological safety.

## 6. Clinical Translation: Inconsistent Clinical Signals, Delivery, Biomarkers and Safety

Clinical observations do not support a single disease-associated direction for *miR-1* or *miR-133a*. Both are reduced within infarcted myocardium, whereas circulating myomiRs can increase after acute injury because damaged cardiomyocytes release intracellular RNA [102,103]. *miR-133a* is reduced in several AF datasets but is elevated in inflammatory cardiomyopathy tissue and in the circulation during sepsis [132,133,143,144]. These discrepancies may reflect biological compartment, disease etiology and stage, sampling time, comorbid inflammation, medication, hemolysis, and normalization strategy. They should therefore be interpreted as context-dependent signals rather than as mutually exclusive results.

Biomarker development will require multicentre, longitudinal cohorts with standardized blood collection, processing, storage, RNA extraction, hemolysis assessment, and normalization. Assays should distinguish *miR-1-1-3p* and *miR-133a-3p* from related family members, report absolute or externally calibrated concentrations where possible, and stratify patients by phenotype, disease phase, renal function, inflammatory status, and treatment. Panels that combine *miR-1/miR-133a* with established cardiac biomarkers and clinical variables are more likely to be robust than a universal single-miRNA threshold. Prospective validation against prespecified diagnostic or prognostic endpoints is essential before clinical implementation.

Therapeutic delivery presents a different challenge because mimics are required when a protective microRNA is deficient, whereas anti-miRs are required when excessive activity is pathogenic. Local or cell-targeted delivery may reduce systemic exposure: intramyocardial miR-Combo promotes cardiac reprogramming after infarction, RGD-PEG-PLA nanoparticles deliver *miR-133* to infarcted myocardium, and an LNA-modified anti-miR-1 can reverse experimental atrioventricular block [25,76,106]. Chemically stabilized oligonucleotides, biodegradable nanoparticles, ligand-directed uptake, and transient expression systems should be compared directly for cardiac retention, endosomal escape, duration of action, manufacturability, and re-dosability. The delivery platform must be selected for the intended cell type rather than for the heart as a homogeneous organ.

Safety is the principal constraint because each microRNA regulates many transcripts and because both loss and excess can be harmful. Systemic exposure may affect skeletal and smooth muscle, endothelial cells, fibroblasts, immune cells, and off-target organs, while excessive cardiac *miR-1* or *miR-133a* can disturb conduction and repolarization. Development programmes should therefore include dose–response and washout studies, bio distribution, innate-immune activation, liver and kidney toxicity, long-term tumorigenicity, and continuous electrophysiological monitoring. Cell-selective delivery, short exposure windows, and sequence-level off-target screening are preferable to uncontrolled long-term overexpression.

Accordingly, the most realistic near-term applications may be disease-specific biomarker panels and locally delivered, transient interventions in well-defined patient groups. Translation will depend on connecting a reproducible molecular direction to a defined cellular compartment and therapeutic window, rather than assuming that increasing or inhibiting either microRNA will be uniformly beneficial [36].

In sum, clinical translation of *miR-1* and *miR-133a* requires recognition of their compartment-, disease-, and stage-dependent expression patterns. Reliable biomarker development will depend on standardized multicentre validation and their incorporation into disease-specific multimarker panels. Therapeutic applications should prioritize cell-targeted, transient delivery within carefully defined dose ranges to minimize pleiotropic, off-target, immunological, and electrophysiological risks.

## 7. Integrated Synergistic and Antagonistic Actions of *miR-1* and *miR-133*

The biological relationship between *miR-1* and *miR-133* is neither uniformly synergistic nor uniformly antagonistic. Their bicistronic organization synchronizes transcript availability, but their distinct seed sequences distribute regulation across different target networks. During embryogenesis, combined loss of the *miR-1/miR-133a* clusters produces a more severe phenotype than loss of individual loci and derepresses myocardin and the smooth-muscle programme, supporting cooperative buffering of cardiomyocyte identity [60,71]. In the hypertrophic heart, both microRNAs also converge protectively on calcium, MAPK, cytoskeletal, and epigenetic pathways through different targets [95,96]. In these settings, synergy arises because distinct molecular actions reinforce the same biological outcome.

Antagonism emerges when their targets favour opposite state transitions. In ESC-derived progenitors, *miR-1* promotes cardiac differentiation, whereas *miR-133* can preserve a less differentiated state; in the posterior avian heart, *miR-1* enhances and *miR-133a* suppresses RA signalling, producing opposing effects on proliferation and maturation [59,65,66]. Classical myoblast studies similarly showed that *miR-1* favours differentiation, whereas *miR-133* promotes proliferation through distinct targets [58]. These observations represent functional antagonism at the phenotypic level and do not imply that the two microRNAs directly inhibit one another.

The interaction is therefore dose-, stage-, cell-type-, and endpoint-dependent. Physiological expression supports lineage identity and homeostasis, whereas excessive *miR-1* or *miR-133a* can impair ventricular growth or electrical stability. Similarly, *miR-133a* may be antifibrotic yet anti-angiogenic in different cardiac cell populations. Future studies should use factorial dual-miRNA perturbations, single-cell and spatial profiling, and combined transcriptomic/proteomic target mapping to distinguish additive, synergistic, and antagonistic effects quantitatively. This framework reconciles several apparently contradictory findings and cautions against treating bicistronic co-expression as evidence of functional equivalence.

## 8. Conclusions and Future Perspectives

The *miR-1/miR-133* cluster has emerged as one of the most relevant post-transcriptional regulatory networks governing cardiac development and cardiovascular homeostasis. Experimental evidence obtained from multiple vertebrate models has consistently demonstrated that both microRNAs are indispensable for proper cardiogenesis, controlling key processes such as cardiac progenitor specification, cardiomyocyte proliferation, differentiation, maturation, and electrophysiological stability. Importantly, their functions extend beyond embryonic development, as dysregulation of *miR-1* and *miR-133* contributes to several pathological processes underlying heart failure, including cardiac hypertrophy, fibrosis, angiogenesis imbalance, inflammation, and arrhythmogenesis.

Although *miR-1* and *miR-133* are frequently studied as individual molecules, the available evidence suggest that they should be considered components of an integrated regulatory network. Their coordinated expression from common bicistronic clusters, together with their complementary and occasionally antagonistic biological functions, enables the fine-tuning of complex molecular pathways required for both cardiac development and disease progression. This duality highlights the importance of understanding not only their individual targets but also their dynamic interactions within broader gene regulatory circuits.

Despite significant advances, several fundamental questions remain unresolved. Most studies have focused on the canonical role of *miR-1* and *miR-133* as cytoplasmic post-transcriptional repressors. However, recent evidence demonstrating a nuclear function of *miR-133a* in the epigenetic regulation of *Dnmt3b* suggests that these microRNAs may exert previously unrecognized transcriptional and chromatin-remodelling activities. Whether similar non-canonical mechanisms exist for *miR-1*, and to what extent such nuclear functions contribute to cardiac development, maturation, and disease, remains largely unknown.

Another intriguing aspect concerns the marked evolutionary differences in the expression pattern of these microRNAs. Whereas *miR-1* and *miR-133* are robustly expressed in the developing hearts of amniotes, their cardiac expression appears absent or substantially reduced during embryonic development in zebrafish and *Xenopus*. Understanding the molecular and evolutionary basis of these differences may uncover alternative regulatory mechanisms controlling vertebrate cardiogenesis and could provide valuable insights into species-specific regenerative capacities.

From a translational perspective, the ability of *miR-1* and *miR-133* to promote direct cardiac reprogramming has positioned these microRNAs among the most promising candidates for regenerative cardiovascular therapies. Nevertheless, several challenges must be overcome before their clinical implementation becomes feasible, including efficient and tissue-specific delivery, long-term safety, dosage optimization, and minimization of off-target effects. Likewise, although altered expression of *miR-1* and *miR-133* has been consistently associated with multiple cardiovascular disorders, additional studies are required to validate their utility as reliable biomarkers for disease diagnosis, prognosis, and therapeutic monitoring.

The increasing availability of single-cell transcriptomics, spatial transcriptomics, epigenomic profiling, and multi-omics approaches offer unprecedented opportunities to further dissect the biological functions of the *miR-1/miR-133* cluster. These technologies will enable the identification of cell type-specific targets and regulatory networks that remain masked in bulk tissue analyses. Furthermore, growing evidence indicates extensive interactions between microRNAs, long non-coding RNAs, and circular RNAs, suggesting that the biological impact of the *miR-1/miR-133* cluster should be interpreted within the context of a broader non-coding RNA regulatory landscape.

In conclusion, the *miR-1/miR-133* cluster represents a pivotal molecular hub linking cardiac development, cellular homeostasis, tissue remodelling, and heart failure progression. A deeper understanding of its canonical and non-canonical mechanisms of action, together with the integration of emerging multi-omics technologies and translational approaches, will undoubtedly advance our knowledge of cardiovascular biology and facilitate the development of innovative therapeutic strategies for cardiac disease.

## Data Availability

No new data were created or analyzed in this study. Data sharing is not applicable to this article.
